# Predicting Pharmacodynamic Drug-Drug Interactions through Signaling Propagation Interference on Protein-Protein Interaction Networks

**DOI:** 10.1371/journal.pone.0140816

**Published:** 2015-10-15

**Authors:** Kyunghyun Park, Docyong Kim, Suhyun Ha, Doheon Lee

**Affiliations:** 1 Department of Bio and Brain Engineering, KAIST, 291 Daehak-ro, Yuseong-gu, Daejeon 305–701, Republic of Korea; 2 Bio-Synergy Research Center, KAIST, 291 Daehak-ro, Yuseong-gu, Daejeon 305–701, Republic of Korea; Universidad de Malaga, SPAIN

## Abstract

As pharmacodynamic drug-drug interactions (PD DDIs) could lead to severe adverse effects in patients, it is important to identify potential PD DDIs in drug development. The signaling starting from drug targets is propagated through protein-protein interaction (PPI) networks. PD DDIs could occur by close interference on the same targets or within the same pathways as well as distant interference through cross-talking pathways. However, most of the previous approaches have considered only close interference by measuring distances between drug targets or comparing target neighbors. We have applied a random walk with restart algorithm to simulate signaling propagation from drug targets in order to capture the possibility of their distant interference. Cross validation with DrugBank and Kyoto Encyclopedia of Genes and Genomes DRUG shows that the proposed method outperforms the previous methods significantly. We also provide a web service with which PD DDIs for drug pairs can be analyzed at http://biosoft.kaist.ac.kr/targetrw.

## Introduction

According to the Centers for Disease Control and Prevention, National Center for Health Statistics, from 1999–2000 to 2007–2008, the percentage of the US population taking two or more prescription drugs increased from 25.4% to 31.2% [1]. In addition, the percentage taking five or more prescription drugs increased from 6.3% to 10.7% [1]. Interestingly, in 2007–2008, less than 10% of children under 12 years of age took two or more prescription drugs, and only 1% took five or more prescription drugs [1]. On the other hand, in the same period more than 76% of older Americans aged 60 and over took two or more prescription drugs and 37% took five or more prescription drugs [1]. The survey results illustrate that more people take multiple medications with passing time and older people take more of them than younger people. One consequence of the growing use of multiple prescription drugs is the potential for interaction between drugs, which can lead to serious side effects. In practice, critical drug-drug interactions (DDIs) have resulted in the withdrawal of drugs from usage [2, 3]. For example, mibefradil and cerivastatin were withdrawn from the US market due to adverse effects from severe DDIs [3, 4]. In addition to such potential health risks, the pharmaceutical industry invests much time and money in drug development. Therefore, identifying possible DDIs at an early stage of drug development is crucial for the safety of patients and the success of a drug.

DDI is defined as a change in efficacy of a drug by the co-administration of two or more drugs [5]. DDIs are categorized into pharmacodynamic and pharmacokinetic interactions [6]. The pharmacodynamic DDI (PD DDI) occurs when one drug interferes with another drug at a target site or affects another protein within the same pathway [7, 8]. One drug can alter the effects of another drug if they have same signaling pathway [8]. It is interference with pharmacodynamics by affecting mechanism of action of the drug without altering pharmacokinetics. The pharmacokinetic DDI (PK DDI) occurs when one drug changes the absorption, distribution, metabolism, or excretion property of another drug [7].

To analyze a large number of DDI candidates, computational approaches have been developed with methods that can predict possible DDIs using various types of drug information (e.g., chemical structure, target proteins, and side effects) [6, 9]. Here, we categorize those previous computational approaches into similarity-based, knowledge-based, and mechanism-based methods. The similarity-based method assumes that drugs with similar properties will have similar DDIs. Based on this assumption, Vilar *et al*. measured drug similarity using the Tanimoto coefficient between the interaction profile fingerprints of drug pairs [9]. Cheng *et al*. integrated chemical, side effect, therapeutic, and genomic properties and constructed a database of adverse drug events [10–13]. Li *et al*. developed a Bayesian network integrating drug similarity using drug molecular and pharmacological phenotypes [14]. In addition, Gottlieb *et al*. used seven kinds of methods for calculating drug similarity (e.g., chemical-based, ligand-based, side-effect based, annotation-based, sequence-based, closeness-based in a protein-protein interaction (PPI) network, and Gene Ontology-based similarity) [6]. The knowledge-based method uses the literature and the FDA Adverse Event Reporting System (FAERS) to predict DDIs [15, 16]. Segura-Bedmar *et al*. predicted the DDIs from text information on drugs and their interactions in DrugBank using a shallow linguistic kernel [16]. Tatonetti *et al*. identified drug effects and interactions using FAERS. The mechanism-based method predicts the PD DDIs using drug target associations in molecular level. Yildirim *et al*. constructed a drug network by connecting drugs if they shared target proteins [17]. Recently, Huang *et al*. developed a target-center system for each drug, which consists of drug targets and their first neighbors in the PPI network and human tissue gene expression [18]. To predict as well as prevent the PD DDIs, it is necessary to identify the mechanism of the interactions. Therefore, this study focused on the mechanism-based approaches.

Signaling starting from drug targets would propagate through the PPI network because the PPI network transfers the biological function through the assembly of a protein signal cascade [19, 20]. Therefore, PD DDIs could occur by affecting the close interference as well as distant interference between drug effects. However, most of the previous mechanism-based approaches have considered only the close interference. The aim of this study is to predict PD DDIs by considering both close and distant interferences of signaling propagation through the PPI network.

## Materials and Methods

Our research framework can be categorized into three parts (S1 Fig). First, in the data preprocessing part, we prepared the DDIs, drug-target associations, and PPI network from various databases [21–27]. Second, in the algorithm part, we applied our method and the shortest path length average (SPA) method using drug-target associations and the PPI network. Last, in the validation and analysis part, we compared the area under a receiver operating characteristic curve (AUC) and fold enrichment of our method with those of the previous methods. In addition, the PD DDI candidates were analyzed by drug side effects and significant PD DDI-associated genes were analyzed by functional annotation analysis.

### Overview of an algorithm for predicting PD DDIs

**Fig 1:**
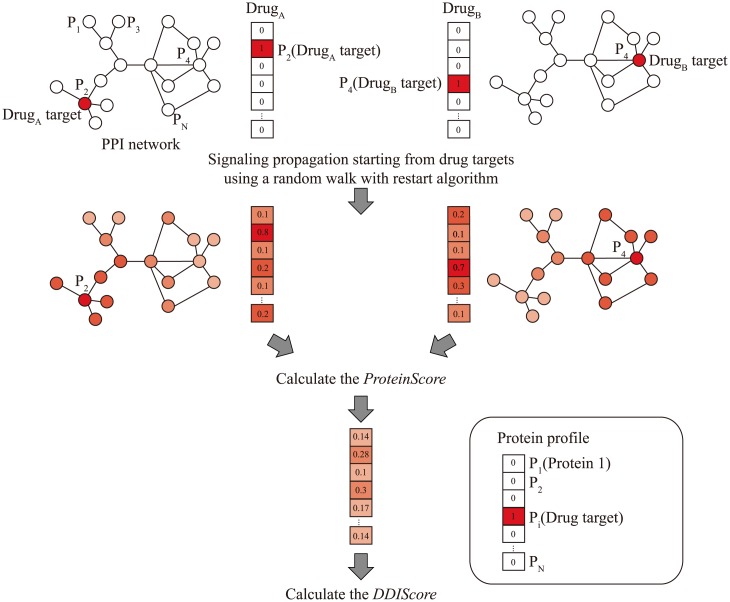
Overview of the algorithm for predicting PD DDIs. The signaling propagation starting from drug targets (Drug_A_: Protein 2 and Drug_B_: Protein 4) through the PPI network is simulated by RWR algorithm. The *ProteinScore* is defined as the values of the protein profile of drug pairs after finishing the simulation of RWR algorithm. The *DDIScore* is calculated by the summation of the *ProteinScore* of all proteins. The *DDIScore* represents the interference of signaling propagation through PPI network between drugs.

A random walk with restart (RWR) algorithm can simulate that the random walker from its nodes (proteins) randomly transits to the neighbor nodes on the PPI network starting from drug targets (Fig 1) [28]. The probability of being at each protein was calculated by the RWR algorithm. We took the probability of proteins to represent the influence initiated by drug targets on the PPI network. The RWR algorithm simulates the random walker until the saturation of probability for all of the proteins on the PPI network. Next, we calculated the *ProteinScore*, which represents the overlapping influence on the same proteins from two drugs. In addition, we calculated the *DDIScore* by summation of the *ProteinScore* of all proteins. We used the *DDIScore* as the possibility measurement for the occurrence of PD DDIs between the drugs.

### Data sets

4,085 drugs in DrugBank were used in this study [27]. 14,516 drug-target associations were used in DrugBank [27]. We used 281 pathways from Kyoto Encyclopedia of Genes and Genomes (KEGG) [25]. Previous studies used non- cytochrome P450 (CYP)-related DDIs that are not metabolized by the same CYP between drugs in known DDIs and DDIs from a semi-automatic text mining method as PD DDIs [6, 18]. We thought that their PD DDIs would include many false-positive PD DDIs. The PD DDI occurs when one drug interferes with another drug at a target site or affects another protein within the same pathways [7, 8]. To make more precise PD DDIs, we used known DDIs as PD DDIs when their targets were same or within the same pathway. The known DDIs were downloaded from DrugBank and KEGG DRUG [25, 27]. We used two sources of PD DDIs, namely, 2,348 PD DDIs and 13,357 PD DDIs from DrugBank and KEGG DRUG, respectively [25, 27]. The KEGG identifier (ID) was converted to DrugBank ID using their data (ftp://ftp.genome.jp/pub/kegg/medicus/drug/drug). We used three human protein interaction networks, Human Protein Reference Database (HPRD) (9,117 proteins and 36,248 interactions), Interologous Interaction Database (I2D) (9,478 proteins and 43,593 interactions), and IntAct (10,942 proteins and 58,303 interactions) from Human Integrated Protein-Protein Interaction rEference (HIPPIE) [21–24, 26]. In addition, we used the integrated PPI network (12,492 proteins and 82,694 interactions) by HPRD, I2D, and IntAct from HIPPIE [21–24, 26]. A rule format based on our previous work was used to describe the PPI network [29]. The DDI side effects were downloaded from TWOSIDES (http://www.pharmgkb.org/downloads/) [15]. In addition, the drug side effects were downloaded from SIDER (http://sideeffects.embl.de/download/) [30]. To identify associations between drugs, we used STITCH that provides the interactions of chemicals from various data sources such as the result of text mining and the knowledge of the other associated databases [31]. The STITCH drug ID in TWOSIDES and SIDER was converted to the DrugBank ID using the STITCH file (chemical.sources.tsv.gz) [31].

### An algorithm for predicting PD DDIs

The RWR algorithm could simulate the signaling propagation starting from drug targets through the PPI network. We assumed that the probability of being at each protein according to the RWR algorithm is the value of the influence starting from drug targets on the PPI network. The probability vector of being each node at time step *t*+1 by RWR algorithm was defined as

$$p\left(t+1\right)=\left(1-r\right)W^{T}\;p\left(t\right)+rp\left(0\right)$$ (1)

where *r* represents the restarting probability of the random walker at each time step, *W* represents the normalized adjacency matrix of the PPI network, *p(t)* is the probability vector of being each node at time step *t*, and *p*(0) represents initial probability vector [32–34].

The initial probability of drug targets among proteins on the PPI network is 1 and the others are 0. In addition, the RWR algorithm was simulated until the sum of the absolute differences of each protein probability between the previous step and current step was less than 1.0E-5 with the restarting probability of 0.7. The *ProteinScore* and *DDIScore* are defined as

$$ProteinScore_{i}\left(Drug_{A},Drug_{B}\right)=\sqrt{V_{i}\left(Drug_{A}\right)\times V_{i}\left(Drug_{B}\right)}$$ (2)

$$DDIScore\left(Drug_{A},Drug_{B}\right)=\sum_{i=1}^{N}ProteinScore_{i}\left(Drug_{A},Drug_{B}\right)$$ (3)

where *i* represents the index of proteins, *v*_*i*_ *(Drug*_*A*_ *)* represents the probability of being at the protein *i* of *Drug*_*A*_ after the finishing simulation of RWR algorithm, and *N* represents the number of proteins on the PPI network.

The *ProteinScore* represents the overlapping influence on the same proteins between drugs. In addition, the *DDIScore* represents the interference score between drugs as determined by the summation of the *ProteinScore* of all proteins.

### Comparison with previous methods

In this study, we evaluated performance using two DDI databases, DrugBank and KEGG DRUG [25, 27], and three PPI network databases, HPRD, I2D, and IntAct in HIPPIE [21–24, 26]. For evaluation, our method was compared with the SPA method used in the Gottlieb *et al*. study and the target-center system in the Huang *et al*. study [6, 18].

Gottlieb *et al*. used SPA among the targets of drugs on the PPI network, one of various similarity methods [6]. The SPA measured how close drug targets were on the PPI network. Considering this, we thought that the SPA method does not exploit the distant interference of signaling propagation through the PPI network between drugs. Therefore, the comparison between our method and the SPA method could identify the importance of distant interference of signaling propagation for predicting PD DDIs. The SPA was defined as

$$SPA(Drug_{A},Drug_{B})=\frac{\sum_{\alpha \in T\left(Drug_{A}\right)}\sum_{\beta \in T\left(Drug_{B}\right)}S\left(p^{\alpha },p^{\beta }\right)}{\left|T\left(Drug_{A}\right)\right|\times \left|T\left(Drug_{B}\right)\right|}$$ (4)

T(Drug)={x|x is target protein of Drug} (5)

$$S\left(p,p^{\prime }\right)=\{\begin{array}{c} \text{1}\\ A\cdot e^{-D\left(p,p^{\prime }\right)} \end{array}\begin{array}{c} \text{if }p\text{ is equal to }p^{\prime }\\ \text{otherwise} \end{array}$$ (6)

where *A* is assigned a value of 0.9 e, *p* and *p*′ are two proteins on the PPI network, and *D*(*p*, *p*′) is the shortest path length between *p* and *p*′ on the PPI network.

In addition, our study was compared with Huang *et al*.*’s* method [18]. They used the local topology of the PPI network and gene expression profiles across human tissues. We downloaded the S-score from the study, which describes the connection between the target-centered system of two drugs, from their website (http://www.picb.ac.cn/hanlab/DDI) [18]. To fairly compare our method with Huang *et al*.*’s* method, we used the same PD DDI candidates (217,743 PD DDIs) in both of them.

### Evaluation methods of predicted PD DDIs

For performance evaluation, the AUC of our method was compared with that of previous methods. We identified the performance of our method for single-target drugs and multi-target drugs. In 14 Anatomical Therapeutic Chemical (ATC) codes, we identified whether our method’s performance was consistent with their acting therapeutic and chemical characteristic organs or systems. To additionally analyze our result, we used known DDIs in DrugBank and KEGG DRUG and DDI side effects in TWOSIDES. We measured the fold enrichment of the top 5% of PD DDI candidates using known DDIs. In addition, we identified the fold enrichment of the top 5% of PD DDI candidates using the DDIs with the highest confidence score of DDI side effects in TWOSIDES [15]. The fold enrichment was defined as

$$Fold\;enrichment=\frac{m/n}{M/N}$$ (7)

where *m* is the number of known DDIs (or DDIs with the highest confidence score in TWOSIDES) in the top 5% of PD DDI candidates, *n* is the number of the top 5% of PD DDI candidates, *M* is the number of known DDIs (or DDIs with the highest confidence score in TWOSIDES) of all PD DDI candidates, and *N* is the number of all PD DDI candidates.

The PD DDIs with the highest scores in our method were analyzed by TWOSIDES, SIDER, and STITCH [15, 30, 31]. The Jaccard index of side effects of a drug pair was defined as below.

$$SEsim\left(Drug_{A},Drug_{B}\right)=\frac{\left|SE\left(Drug_{A}\right)\cap SE\left(Drug_{B}\right)\right|}{\left|SE\left(Drug_{A}\right)\cup SE\left(Drug_{B}\right)\right|}$$ (8)

SE(Drug)={x|x is no placebo and frequent effects of Drug in SIDER} (9)

We identified the significant PD DDI-associated genes and PD DDI candidates using the empirical p-value. For estimating the empirical p-value, we determined the empirical distribution of *ProteinScore* from randomly selected proteins and randomly selected drug pairs, repeating 100,000 times. In addition, to make the empirical distribution of the *DDIScore* as well, we also measured the *DDIScore* from randomly selected drugs for each protein, repeating 100,000 times. The selected significant genes (empirical p-value<1.0E-4) were analyzed by functional annotation analysis using DAVID [35].

## Results

### Performance evaluation

**Fig 2:**
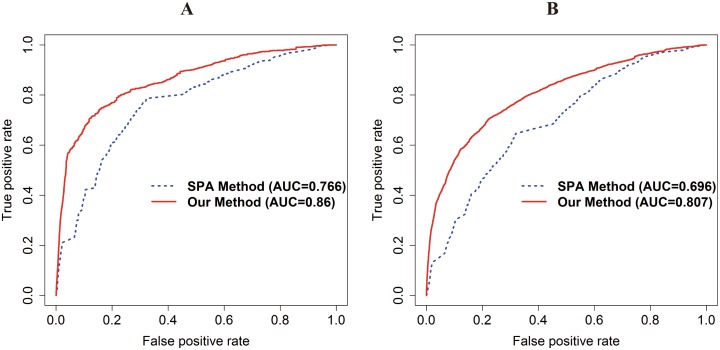
A receiver operating characteristic (ROC) curve with AUC in DrugBank and KEGG DRUG for comparison with the SPA method using integrated PPI network. The red and blue lines represent our method and the SPA method, respectively. (A) The ROC curve with the AUC of our method and the SPA method in DrugBank. (B) The ROC curve with the AUC of our method and the SPA method in KEGG DRUG. The AUC of our method and the SPA method in each DDI database are 0.86 and 0.766 in DrugBank, 0.807 and 0.696 in KEGG DRUG.

Our method and SPA method are evaluated by the same candidate drug pairs in each PPI network (HPRD: 739,936 PD DDIs; I2D: 735,078 PD DDIs; IntAct: 591,328 PD DDIs; integrated PPI network (HPRD, I2D, and IntAct): 779,376 PD DDIs). The AUC of our method is 0.86 and 0.807 and that of the SPA method is 0.766 and 0.696 using the integrated PPI network in DrugBank and KEGG DRUG, respectively (Fig 2A and 2B). The fold enrichment of the top 5% of the PD DDI candidates of our method and the SPA method is 11.573 and 4.492 in DrugBank and 7.946 and 3.082 in KEGG DRUG, respectively. In addition, in DrugBank and KEGG DRUG, our method has also higher accuracy than the SPA method using HPRD, I2D, and IntAct, respectively (S2–S4 Figs).

**Fig 3:**
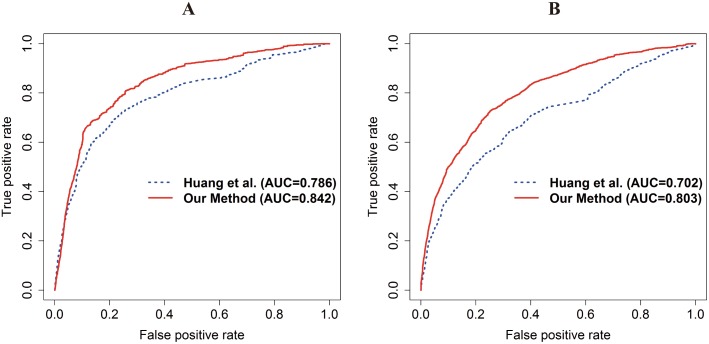
ROC curve with AUC in DrugBank and KEGG DRUG for comparison with Huang *et al*.*’s* method using the integrated PPI network. The red and blue lines represent our method and Huang *et al*.*’s* method, respectively. (A) The ROC curve with the AUC of our method and Huang *et al*.*’s* method in DrugBank. (B) The ROC curve with the AUC of our method and Huang *et al*.*’s* method in KEGG DRUG. The AUC of our method and Huang *et al*.*’s* method in each DDI database are 0.842 and 0.786 in DrugBank and 0.803 and 0.702 in KEGG DRUG. The performance of our method generally outperforms that of Huang *et al*.*’s* method.

For fair comparison with the target-center system in the Huang *et al*. study [18], we used the same PD DDI candidates (1,127 drugs and 217,743 PD DDIs) because different PD DDI candidates would cause a bias of performance. The AUC of our method using the integrated PPI network was compared with that of the S-score of the Huang *et al*. study. The AUC of our method and Huang *et al*.*’s* method is 0.842 and 0.786 in DrugBank and 0.803 and 0.702 in KEGG DRUG, respectively (Fig 3A and 3B). In addition, the fold enrichment of the top 5% of the PD DDI candidates of our method and Huang *et al*.*’s* method is 7.291 and 6.583 in DrugBank and 6.339 and 4.6 in KEGG DRUG, respectively. From these results, we concluded that our method outperforms the SPA method and Huang *et al*.*’s* method.

### Evaluation of predicted PD DDIs using the number of drug targets and ATC codes

**Fig 4:**
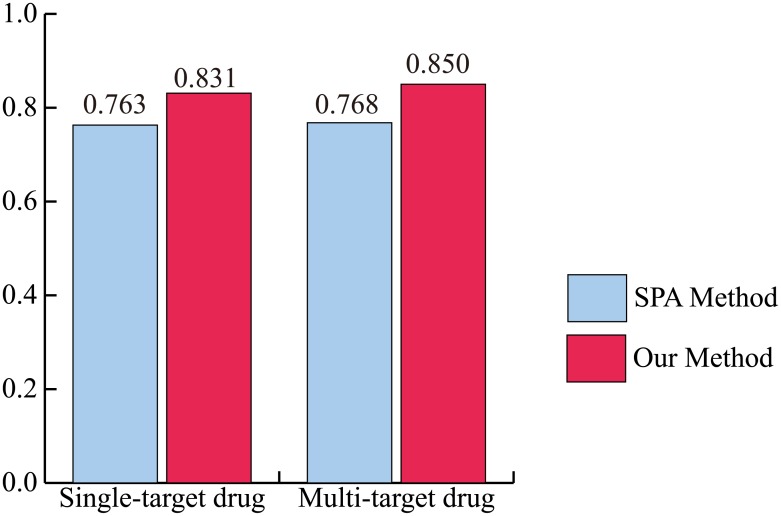
The AUC of our method and the SPA method for single-target drug and multi-target drug categories. The AUC of our method outperforms that of the SPA method in both single-target drug and multi-target drug categories.

**Fig 5:**
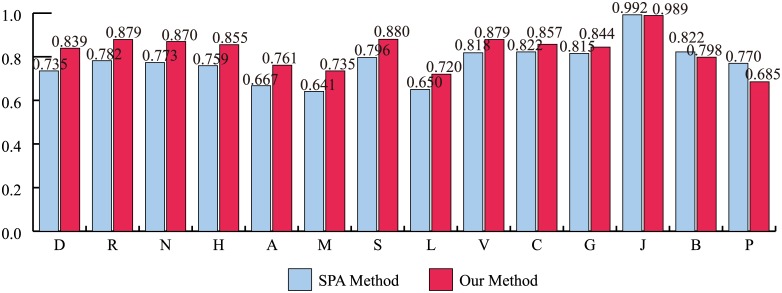
The AUC of our method and the SPA method for 14 ATC codes. For all of the ATC codes except J (antiinfectives for systemic use), B (blood and blood forming organs), and P (antiparasitic products, insecticides and repellents) codes, our method outperforms the SPA method.

Recently, the paradigm in drug discovery has shifted from single-target drugs to multi-target drugs [36]. Therefore, it becomes necessary to identify the performance of PD DDIs for multi-target drugs. To evaluate the performance of our method for this purpose, the drugs were first categorized into single-target drugs and multi-target drugs by the number of drug targets. Fig 4 shows that our method has more accurate predictions than the SPA method for both of the single-target drug and multi-target drug categories using the integrated PPI network. For single-target drugs, the AUC of our method and SPA method is 0.831 and 0.763. For multi-target drugs, the AUC of our method and SPA method is 0.850 and 0.768. In addition, to evaluate performance robustness, the drugs were categorized into 14 ATC codes. We also identified the performance of our method and the SPA method for these 14 ATC codes using the integrated PPI network. The result shows that our method has higher accuracy than the SPA in overall ATC codes (Fig 5). The “B” code (the first part of the ATC code) represents blood and blood forming organs. Our method and the SPA method have a similar performance. In this case, PD DDIs could occur by the close interference between drugs because the drug might directly bind to disease-associated proteins. For example, lepirudin (B01AE02) is used for the treatment of heparin-induced thrombocytopenia. The drug binds directly to thrombin to prevent thrombus [27]. The “P” code represents antiparasitic products, insecticides and repellents. It should be noted that the comparison between our method and the SPA method might not be appropriate for non-human of drug target organisms because human protein interactions in HIPPIE were only used in this study [22]. For example, the target organisms of metronidazole (P01AB01) are bacteria and protozoa in DrugBank [27]. Nonetheless, from these results, we propose that our method would be an effective approach for predicting PD DDIs in both single-target drugs and multi-target drugs as well as various ATC codes.

### Evaluation of predicted PD DDIs using known DDIs and DDI side effects

We measured fold enrichment with our method and the SPA method using both types of known DDIs including both PD and PK DDIs in DrugBank and KEGG DRUG as a positive gold standard because using the selected PD DDIs as the positive gold standard could cause a bias of performance. The fold enrichment of the top 5% of the PD DDI candidates of our method and the SPA method is 3.522 and 1.918 in DrugBank and 3.913 and 1.703 in KEGG DRUG, respectively. In addition, we also calculated the fold enrichment of DDI side effects with the highest confidence score in TWOSIDES because unreported DDIs could cause DDI side effects [15]. The fold enrichments of the top 5% of the PD DDI candidates of our method and the SPA method are 2.252 and 0.827, respectively. From these results, we note that the known DDIs and DDI side effects in the top 5% of the PD DDI candidates of our method are more enriched than those of the SPA method.

### Case study

For a case study, we selected ziprasidone-quetiapine, olanzapine-quetiapine, and aripiprazole-quetiapine interactions, which had the highest PD DDIs scores. Their scores are the same because ziprasidone, olanzapine, and aripiprazole have identical target proteins in DrugBank [27]. Ziprasidone, olanzapine, aripiprazole, and quetiapine are used for the treatment of schizophrenia in DrugBank [27]. Ziprasidone-quetiapine interaction is reported in DrugBank and KEGG DRUG and olanzapine-quetiapine and aripiprazole-quetiapine interactions are reported in KEGG DRUG [25, 27]. We analyzed these interactions by using TWOSIDES, SIDER, and STITCH [15, 30, 31]. Olanzapine-quetiapine and aripiprazole-quetiapine interactions have significant DDI side effects (p-value: 1.08E-249 and 9.65E-123) in TWOSIDES. Campillos *et al*. suggested that drug targets could be identified by similarity of side effects [37]. Therefore, we identified whether drugs that have similar side effects would have also the same target proteins, which is one of the mechanisms causing PD DDIs. The Jaccard index of ziprasidone-quetiapine, olanzapine-quetiapine, and aripiprazole-quetiapine interactions using the side effects of each drug in SIDER is 0.288 (p-value: 1.25E-27), 0.390 (p-value: 1.04E-41), and 0.317 (p-value: 9.46E-35). In practice, 25 of 26 targets of quetiapine are the same targets as those of ziprasidone, olanzapine, and aripiprazole in DrugBank (S1 Table) [27]. In STITCH, a score greater than 0.7 is considered to be a high confidence score based on their criteria [31]. The confidence scores of ziprasidone-quetiapine, olanzapine-quetiapine, and aripiprazole-quetiapine interactions are 0.798, 0.83, and 0.77, respectively [31]. In addition, we identified 306 PD DDI-associated genes (empirical p-value<1.0E-4) from a permutation test (S2 Table). Table 1 shows the significant KEGG pathways (False Discovery Rate (FDR) <5.0E-3) based on a functional annotation analysis using the Database for Annotation, Visualization and Integrated Discovery (DAVID), except for the O75970 protein, which had no mapped identifier in DAVID [35]. From these results, we suggest that the interfered pathways could change the efficacy of the drugs.

**Table 1:** The significant KEGG pathway lists (FDR<5.00E-03) from 305 PD DDI-associated genes (except O75970 protein) using DAVID.

KEGG pathways	p-value	FDR
**Neuroactive ligand-receptor interaction**	4.19E-42	4.68E-39
**Calcium signaling pathway**	6.66E-28	7.43E-25
**Gap junction**	1.39E-12	1.56E-09
**Long-term depression**	6.39E-12	7.14E-09
**Long-term potentiation**	4.05E-09	4.52E-06
**Vascular smooth muscle contraction**	7.57E-09	8.44E-06
**Chemokine signaling pathway**	4.38E-08	4.89E-05
**Glioma**	8.62E-08	9.62E-05
**Neurotrophin signaling pathway**	2.36E-07	2.64E-04

We suggest that these significant pathways affected the drug effects through signaling propagation interference between drugs.

## Discussion

Signaling transduction starting from the drug targets propagates to their neighbor nodes through the PPI network [19, 20]. Therefore, PD DDIs could occur by both close and distant interferences of signaling propagation through the PPI network. However, previous methods for predicting PD DDIs have only the close interference. The SPA method measured the shortest path length between the targets of drugs on the PPI network [6]. The shortest path length only considers how close drug targets are on the PPI network. In addition, the target-centered system in Huang *et al*. study used the PPI sub-network consisting of drug targets and their first neighbors and human tissue gene expression [18]. They calculated a system connection score (S-score) between drugs for predicting PD DDIs. It also considers close interference by reflecting tightness of connections between two target-centered systems. In this study, we propose that both close and distant interferences of signaling propagation through the PPI network are important features for predicting potential PD DDIs.

Our method exploited the global topology of the PPI network [28]. The score of drug pairs was calculated by the amount of overlapped signaling propagation starting from the drug targets through the PPI network. For evaluation, the performance of our method was compared with that of the SPA method and a recent PD DDI prediction method [6, 18]. We identified that the AUC and fold enrichment of our method outperformed those of the SPA method and the recent method.

Developing multi-target drugs is a new paradigm in drug discovery [36]. Recently, Zheng *et al*. proposed a weighted ensemble similarity algorithm to predict the drug-target direct interactions [38]. Therefore, we evaluated the performance of our method for multi-target drugs. Drugs were categorized into single-target drugs and multi-target drugs by the number of drug targets. The AUC of our method outperformed the SPA method in each category. In addition, we showed that the top 5% of the PD DDI candidates of our method had a higher fold enrichment of DDI side effects than did the SPA method. DDI side effects can occur by an unknown DDI. Therefore, the results indicated that our method could predict novel PD DDIs in the new paradigm in drug discovery.

DDIs are conventionally categorized into PD DDIs and PK DDIs [6]. As DDIs are predicted by the pharmacodynamic properties, it is hard to predict PK DDIs. In addition, PD DDIs could have antagonistic, synergistic, or additive effects [8, 18]. However, in this study, our method could not classify the effect of PD DDIs. In the future, we are going to solve these limitations and improve our method.

Our method more accurately predicts PD DDIs than previous methods. In addition, PD DDI-associated genes from our method can be used to interpret the causes of PD DDIs and prevent them. Therefore, we expect that our method will be helpful in predicting and preventing PD DDIs at an early stage of drug development.

## Supporting Information

S1 Fig Research framework. Our research framework can be categorized into three parts such as data preprocessing part, algorithm part, and validation and analysis part.
(EPS)

S2 Fig ROC curve with AUC in DrugBank and KEGG DRUG for comparison with the SPA method using HPRD. The red and blue line represents our method and SPA method. (A) The ROC curve with the AUC of our method and SPA method in DrugBank. (B) The ROC curve with the AUC of our method and SPA method in KEGG DRUG. The AUC of our method and SPA method in each PD DDI database: 0.863 and 0.783 in DrugBank, 0.812 and 0.719 in KEGG DRUG. The result showed that our method outperformed the SPA method.
(EPS)

S3 Fig ROC curve with AUC in DrugBank and KEGG DRUG for comparison with the SPA method using I2D. The red and blue line represents our method and SPA method. (A) The ROC curve with the AUC of our method and SPA method in DrugBank. (B) The ROC curve with the AUC of our method and SPA method in KEGG DRUG. The AUC of our method and SPA method in each PD DDI database: 0.856 and 0.773 in DrugBank, 0.81 and 0.714 in KEGG DRUG. The result showed that our method outperformed the SPA method.
(EPS)

S4 Fig ROC curve with AUC in DrugBank and KEGG DRUG for comparison with the SPA method using IntAct. The red and blue line represents our method and SPA method. (A) The ROC curve with the AUC of our method and SPA method in DrugBank. (B) The ROC curve with the AUC of our method and SPA method in KEGG DRUG. The AUC of our method and SPA method in each PD DDI database: 0.832 and 0.721 in DrugBank, 0.748 and 0.616 in KEGG DRUG. The result showed that our method outperformed the SPA method.
(EPS)

S1 Table Target proteins of ziprasidone, olanzapine, aripiprazole, and quetiapine. 25 of 26 targets of quetiapine are the same targets as those of ziprasidone, olanzapine, and aripiprazole in DrugBank.
(XLS)

S2 Table 306 PD DDI-associated genes of ziprasidone-quetiapine, olanzapine-quetiapine and aripiprazole-quetiapine. Three PD DDIs have same PD DDI-associated genes because ziprasidone, olanzapine, and aripiprazole have same target proteins.
(XLS)
